# Usability, Acceptability, and Adherence to an Electronic Self-Monitoring System in Patients With Major Depression Discharged From Inpatient Wards

**DOI:** 10.2196/jmir.6673

**Published:** 2017-04-21

**Authors:** Lise Lauritsen, Louise Andersen, Emilia Olsson, Stine Rauff Søndergaard, Lasse Benn Nørregaard, Philip Kaare Løventoft, Signe Dunker Svendsen, Erik Frøkjær, Hans Mørch Jensen, Ida Hageman, Lars Vedel Kessing, Klaus Martiny

**Affiliations:** ^1^ Psychiatric Center Copenhagen Rigshospitalet University of Copenhagen Copenhagen Denmark; ^2^ Monsenso App Copenhagen Denmark; ^3^ Rehfeld Medical Copenhagen Denmark; ^4^ Department of Computer Science Copenhagen University Copenhagen Denmark

**Keywords:** depressive disorder, major, electronic monitoring, graph drawing, sleep, self-assessment, observational study, inpatients, patient participation, chronotherapeutics

## Abstract

**Background:**

Patients suffering from depression have a high risk of relapse and readmission in the weeks following discharge from inpatient wards. Electronic self-monitoring systems that offer patient-communication features are now available to offer daily support to patients, but the usability, acceptability, and adherence to these systems has only been sparsely investigated.

**Objective:**

We aim to test the usability, acceptability, adherence, and clinical outcome of a newly developed computer-based electronic self-assessment system (the Daybuilder system) in patients suffering from depression, in the period from discharge until commencing outpatient treatment in the Intensive Outpatient Unit for Affective Disorders.

**Methods:**

Patients suffering from unipolar major depression that were referred from inpatient wards to an intensive outpatient unit were included in this study before their discharge, and were followed for four weeks. User satisfaction was assessed using semiqualitative questionnaires and the System Usability Scale (SUS). Patients were interviewed at baseline and at endpoint with the Hamilton depression rating scale (HAM-D17), the Major Depression Inventory (MDI), and the 5-item World Health Organization Well-Being Index (WHO-5). In this four-week period patients used the Daybuilder system to self-monitor mood, sleep, activity, and medication adherence on a daily basis. The system displayed a graphical representation of the data that was simultaneously displayed to patients and clinicians. Patients were phoned weekly to discuss their data entries. The primary outcomes were usability, acceptability, and adherence to the system. The secondary outcomes were changes in: the electronically self-assessed mood, sleep, and activity scores; and scores from the HAM-D17, MDI, and WHO-5 scales.

**Results:**

In total, 76% of enrolled patients (34/45) completed the four-week study. Five patients were readmitted due to relapse. The 34 patients that completed the study entered data for mood on 93.8% of the days (872/930), sleep on 89.8% of the days (835/930), activity on 85.6% of the days (796/930), and medication on 88.0 % of the days (818/930). SUS scores were 86.2 (standard deviation [SD] 9.7) and 79% of the patients (27/34) found that the system lived up to their expectations. A significant improvement in depression severity was found on the HAM-D17 from 18.0 (SD 6.5) to 13.3 (SD 7.3; *P*<.01), on the MDI from 27.1 (SD 13.1) to 22.1 (SD 12.7; *P*=.006), and in quality of life on the WHO-5 from 31.3 (SD 22.9) to 43.4 (SD 22.1; *P*<.001) scales, but not on self-assessed mood (*P*=.08). Mood and sleep parameters were highly variable from day-to-day. Sleep-offset was significantly delayed from baseline, averaging 48 minutes (standard error 12 minutes; *P*<.001). Furthermore, when estimating delay of sleep-onset (with sleep quality included in the model) during the study period, this showed a significant negative effect on mood (*P*=.03)

**Conclusions:**

The Daybuilder systems performed well technically, and patients were satisfied with the system and had high adherence to self-assessments. The dropout rate and the gradual delay in sleep emphasize the need for continued clinical support for these patients, especially when considering sleep guidance.

## Introduction

### Major Depression

Major depression is estimated by the World Health Organization to top the list of the 20 most financially burdensome disorders in the Western world [1], and a substantial part of these costs cover hospital treatment for patients with severe depression who have long-standing admissions, and who are often readmitted due to relapse [2,3]. Thus, in Denmark, these patients have readmission rates between 10-30% in the months following discharge [4]. Furthermore, there is an increased risk of suicide in the immediate period following discharge [5]. Therefore, new tools and treatments are needed to prevent readmission and suicide after discharge from inpatients wards.

### Electronic Monitoring

Increased access to reliable and fast Internet services and the development of interactive systems have spurred interest in the use of electronic monitoring in medicine [6], including psychiatry, where electronic monitoring is increasingly being used as a clinical tool [7,8]. Existing systems differ in mode of function and complexity. Some systems offer interactive self-help with psychotherapy/psychoeducation [9], some include clinical backup, some are designed for use on smartphones, and others are designed for computers or tablets [10]. A small number of systems use a bidirectional feedback loop between patients and clinicians [11,12], in which data entered by patients can be seen in real-time by the clinician, and responses can occur immediately if needed. These systems make it easier for patients and clinicians to notice changes in conditions over time, be it behavioral changes such as activity or medication, or psychopathological symptoms (eg, mood and sleep). Using these systems, worsening of the condition can be acted upon by patients and clinicians in collaboration, via comonitoring [13]. In general, data entered in these systems are graphically presented to enable patients and researchers/clinicians to visualize relationships between the entered data and development of the measured variables over time.

When used in research, electronic monitoring has a number of advantages: it partly eliminates the need for pen and paper data collection, it makes data immediately available for analyses, and it secures day-to-day information that might otherwise be lost due to cognitive dysfunction that is prevalent in patients with major depression [14]. If the clinical practice can match the opportunities given in interactive systems by supplying feedback, patients will be enabled to make earlier adjustments in treatment and behavior by having an earlier and more focused response to their condition.

High dropout rates are a common problem in electronic monitoring [15], although studies using systems that connect patients and clinicians have higher adherence rates [16]. Some applications have a multitude of options and can be difficult to use for patients with depression. Simplicity and ease of use are considered essential for users [17]. The system that we evaluated in this study (the Daybuilder system) was developed with this in mind, having a reduced number of assessment parameters and a graphical representation that is central in the screen. The Daybuilder system has been developed in close collaboration with users and clinicians in psychiatry [18].

### Summary

In summary, by using interactive electronic monitoring we can: obtain day-to-day information of clinical state; obtain a measure of day-to-day variability; and enable patients and clinicians to discover time-trends and relationships between variables that facilitate early and more focused intervention, via the graphical representation of data over time. A pilot test with the Daybuilder system showed the system to be easy to use and stable [18]. In this study, the aim was to test the usability, acceptability, and adherence to the Daybuilder system in patients suffering from depression who were discharged from inpatient psychiatric wards, and patients were followed for four weeks. We also report relapse, the time courses of mood, sleep, and activity, and the interrelatedness of these factors.

## Methods

### Design

The study design was a single arm observational study. The study consisted of three distinct periods: Phase 1 was the period from inclusion to discharge, Phase 2 was the period from discharge to commencing treatment at the Intensive Outpatient Unit for Affective Disorders (IOA) service, and Phase 3 was the period from the start at IOA to the end of each patient's four-week study period.

### Participants

Patients suffering from unipolar major depression that were referred from inpatient wards to an IOA were asked to participate in the study before they were discharged from psychiatric inpatient wards. The scientific ethical committee for the Capital Region of Copenhagen was informed about the study in writing (Journal nr. H-3-2013-FSP32) and decided that the study did not require review by the committee. The study was approved by Psychiatric Center Copenhagen and the Danish Data Agency (RHP-2013-023, I-Suite number: 02470). The patient identification list was kept under double lock. Baseline procedures consisted of a psychometric assessment and an introduction to the Daybuilder system. Informed consent was obtained from all participants after oral and written information had been given about the study content and possible consequences.

### Eligibility Criteria

Inclusion criteria included: major depression as defined in the Diagnostic and Statistical Manual of Mental Disorders 4^th^edition, and age >18 years. Exclusion criteria included: suicidality (corresponding to a score of 2 or above on the Hamilton depression rating scale [HAM-D_17_] item 3, or if the investigators were unable to assess the degree of suicidality), abuse of alcohol or other substances that could influence the use of the Daybuilder system, bipolar illness, psychotic depression for the last two weeks prior to inclusion, and comorbid dementia or other organic brain damage that could influence the participant’s ability to use the Daybuilder system. Criteria for leaving the study included: patients wishing to leave the study or admittance for a somatic illness that would potentially influence the ability to use the Daybuilder system. Patients were allowed to continue in the study if readmitted to an inpatient psychiatric ward.

### Psychometric Assessment

Sociodemographics were collected through interviews and from case files. Diagnostic confirmation was done by use of the Mini-International Neuropsychiatric Interview instrument [19]. Baseline and endpoint depression severity were assessed by the investigator-administered HAM-D_17_ scale [20] which covers the full spectrum of depression symptoms, the Hamilton six-item subscale (HAM-D_6_) [21] which covers the core symptoms of depression, the Bech-Rafaelsen melancholia scale (MES) which includes items covering symptoms of psychomotor retardation [22], and paper-and-pen self-assessment was done with the Major Depression Inventory (MDI) [23], and the 5-item World Health Organization Well-Being Index (WHO-5) scale [24].

Patients answered semiqualitative questions regarding the usability of the system at baseline and at the endpoint. These questions covered expectations on the use of the system at inclusion, and reflection on experiences with the system at the endpoint. Patients were asked to fill in the System Usability Scale (SUS) at endpoint [25].

### Daybuilder Procedures

A patient-specific profile was created on the Daybuilder webpage for each patient, and each person was assigned a study number and an email address to enable them to log into the system. Patients were instructed on how to use Daybuilder, and how to enter the following variables on all days of the four-week study period: sleep-onset, sleep-offset, number of awakenings at night, quality of sleep, naps (time and duration), mood (morning and evening), activity (number of minutes outside the psychiatric ward, or when discharged as minutes outside their home as an estimate of activity), and medication (whether daily medication were taken or not). Mood and quality of sleep were entered on a Visual Analog Scale (VAS; 0=worst depression/worst sleep ever; 10=no depression/best sleep). Participants were instructed to enter mood scores in the morning and evening. Patients began data entry in the Daybuilder system on the day of inclusion. Study investigators phoned patients weekly to aid with any problems related to Daybuilder and to discuss outcomes of data monitoring, as seen in the Daybuilder graphs. Patients were seen by investigators at a final visit after four weeks. Text messages were used during the project to help patients remember that they had to enter their data. Patients’ data were not seen between telephone calls.

**Figure 1:**
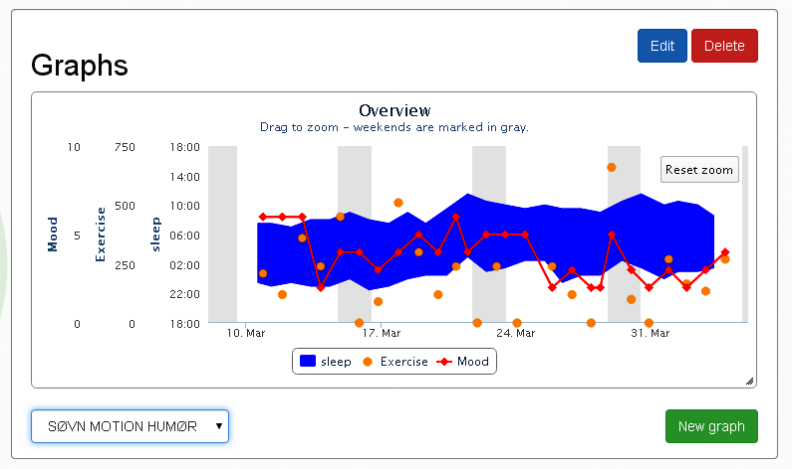
An example of graphic presentation in the Daybuilder application for sleep, mood, and exercise.

All entries into the Daybuilder system were done through the computer (personal computer or Mac), except mood values which could also be entered through short message service (SMS) text messaging. A reminder was sent twice daily over SMS texting to register mood. When entering values in Daybuilder, the system automatically generated a graphic display of all variables to aid understanding of evolving patterns and relationships between variables (eg, between mood, sleep, and activity; see Figure 1).

### Outcomes

The primary outcomes were usability, acceptability, and adherence, which were assessed by SUS scores, semiqualitative questions, and adherence to data entry in the electronic system. Secondary outcomes were changes in self-assessed daily scores of mood, sleep, activity, and medication adherence.

### Statistics

Usability, acceptability, and adherence measures were analyzed on completers, who were defined as patients clinically assessed at endpoint visit. Adherence was calculated as a percentage of data entry days in relation to the planned four-week study period, for each data entry parameter (eg, sleep, mood). Mood, sleep, and activity outcomes were analyzed using available data from all included patients. A mean daily mood score was calculated for those with more than one mood entry per day. Correlation between Hamilton scores and mood scores was calculated using the Pearson correlation procedure.

Daily continuous scale scores that were entered, including sleep scores, were analyzed in a random linear regression model using available data from all included patients with *intercept* and *day* as random effects. Results are given as estimated values, confidence limits (CLs), standard errors (SEs), and *P*-values. For explorative analyses on sleep parameters, the model only included time (day) as a covariate. For explorative analyses on mood the model included time (day), sleep-onset, sleep-offset, sleep quality, activity, and interactions between sleep-onset and day, sleep-offset and day, sleep quality and day, and activity and day. Summary means for sociodemographics, usability, adherence, and acceptability measures are given with standard deviations (SDs). All time points are in the form of hour:minutes.

Based on the paper by Bech et al [26] showing that when comparing MDI depression scores with a VAS scale (0=no depression; 100=worst depression), the cut-off for severe depression on the MDI (scores 31-50, higher scores indicating worse depression) corresponded to a VAS score of 58.4 (MDI=0.49*VAS+2.4). The VAS score used in this study was reversed with 0 as the worst score and 10 as the best, so we translated the VAS score of 58.4 (approximated to 60) to a score of *4 and below* as a signifier of severe depression. The level of statistical significance was set at 5%, and was two-sided. Analyses were performed by SAS software (SAS system for Windows, release 9.4., SAS Inst., Cary, NC, USA).

## Results

### Participants

In total, 230 patients were referred to the IOA in the inclusion period from September 2013 to March 2015. Only 89 patients were asked to participate, as the remaining did not fulfill inclusion criteria or fulfilled exclusion criteria, or were already discharged at the time of screening. A total of 45 patients accepted the invitation to join the study. Sociodemographic data is detailed in Table 1.

**Table 1:** Sociodemographic data.

Sociodemographics	% or mean (SD)
Gender, females	55.6%
Age, years	35.9 (10.8)
Duration of current episode, months	10.2 (11.7)
Number of previous episodes	2.5 (5.7)
Sick leave in current episode	73.3%
Suicide attempt in current episode	8.9%
Number of patients with a self-perceived eliciting factor for actual episode	84.4%
Smoking	22.2%
Electro Convulsive Treatment in current episode	22.2%
Alcohol consumption, number of drinks per month	8.1 (15.6)

Mean age was 35.9 years (SD 10.8). Patients had 2.5 previous episodes of major depression (SD 5.7) and a mean duration of current depression of 10.2 months (SD 11.7). Most patients were on sick leave. Only a few patients had attempted suicide in the current episode before admission. Patients were treated with 1.9 drugs (SD 0.9; range 0-4): antidepressants included Selective Serotonin Reuptake Inhibitors (n=12), Serotonin-Norepinephrine Reuptake Inhibitors (n=15), Noradrenergic and Specific Serotonergic Antidepressants (n=11), mianserin (n=11), Tricyclic Antidepressants (n=15), isocarboxazid (n=1), and agomelatine (n=1); antipsychotics included quetiapine (n=8) and olanzapine (n=2); mood stabilizers included lithium (n=5) and lamotrigine (n=4); benzodiazepines (n=3); hypnotics (n=3); and melatonin (n=2).

### Usability, Acceptability, and Adherence

In total, 76% of enrolled patients (34/45) completed the four-week study; six patients dropped out during study Phase 1, and five patients during study Phase 2. The causes of dropout included worsening of depression for six patients, and miscellaneous nonillness related issues for the remaining five patients. An additional five patients were readmitted to an inpatient ward due to worsening of depression (all in study Phase 2), all of whom continued their self-monitoring and were evaluated at endpoint. Six patients were not discharged on the last day of data entry. Thus, the readmission rate was 13% (5/39). Mean days in study Phase 1 was 6.4 (7.9; range 0-28), study Phase 2 was 7.6 (7.1; range 0-26), and study Phase 3 was 9.9 (9.6; range 0-28; *P*=.14). Electro Convulsive Treatment (ECT) had been used for 22% (10/45) of all patients, 36% (4/11) of the dropouts, and 18% (6/34) of the completers (*P*=.23). Patients who dropped out entered data into Daybuilder for 12.5 days (11.9; range 1-28).

In general, patients found that the Daybuilder system lived up to their expectations; however, when evaluated at endpoint, patients found that they had registered less data than they had anticipated at baseline (Table 2). Fifty-nine percent of patients (20/34) believed that the system could detect a relapse, and 50% of patients (17/34) believed that the system could influence the course of their illness.

Other semiqualitative questions at baseline showed that patients expected the Daybuilder system to enable visualization of their condition, give support and structure, enable positive expectations, or give a hope of recovery. Several additional self-monitoring items were suggested, such as social activity, appetite, meals, anxiety, cognitive function, medication, and side effects. At endpoint, only 50% of patients (17/34) felt that the system had covered their needs for self-monitoring. A total of 33 patients filled in the SUS scale, with a mean value of 86.2 (SD 9.7; range 65-100). Patients not receiving ECT (n=27) had an SUS score of 86.9 (8.9; range 65-100) and patients receiving ECT (n=6) had a score of 82.9 (13.0; range 65-97.5; *P*=.40).

The importance of the weekly phone calls was rated on a scale from 0 to 10 (10=highest importance): 58% of patients (19/33) rated in the interval 8-10, 33% (11/33) in the interval 5-7, and only 9% (3/33) in the interval 0-4. The frequency of phone calls was deemed appropriate by 35% of the patients (12/34). Twenty-six percent of patients (9/34) would have liked more frequent phone calls or a combination of consultation and telephone contact, 12% (4/34) suggested being contacted when the Daybuilder registrations showed signs of deterioration or if data entry was missing, 9% (3/34) felt a need for a more flexible and individualized design that would depend on their mental state, and 18% (6/34) did not answer this question.

**Table 2:** Usability data from semiqualitative questions asked at baseline and endpoint.

Theme		Baseline, % (n=43)	Endpoint, % (n=34)
Did the Daybuilder system live up to expectations?			
	Yes		79 (27)
	No		12 (4)
	Uncertain		9 (3)
	No response		0 (0)
Do you think you will be able to make all registrations/did you make all registrations?			
	Yes	98 (42)	74 (25)
	No	0 (0)	26 (9)
	Uncertain	0 (0)	0 (0)
	No response	2 (1)	0 (0)
Do you expect that self-monitoring of mood will influence your mood/did self-monitoring influence your mood?			
	Yes	44 (19)	32 (11)
	No	37 (16)	65 (22)
	Uncertain	19 (8)	0 (0)
	No response	0 (0)	3 (1)
Is the need for self-monitoring covered in the Daybuilder system?			
	Yes	53 (23)	50 (17)
	No	30 (13)	41 (14)
	Uncertain	12 (5)	0 (0)
	No response	5 (2)	9 (3)
Do you expect that the Daybuilder system can detect/did detect a relapse of depression?			
	Yes	65 (28)	59 (20)
	No	2 (1)	18 (6)
	Uncertain	30 (13)	18 (6)
	No response	2 (1)	6 (2)
Do you expect that the Daybuilder system will influence the course of your illness/did the system influence the course of your illness?			
	Yes	56 (24)	50 (17)
	No	35 (15)	47 (16)
	Uncertain	2 (1)	0 (0)
	No response	7 (3)	3 (1)

Only 9% of patients (3/34) were worried about technical problems and 79% of patients (27/34) found that the system lived up to their expectations. Thirty-two percent of patients (11/34) felt that their self-assessment of mood in the Daybuilder system had influenced their mood, but only one patient felt that it had a negative effect. Adherence with data entry into the Daybuilder application for the 34 completers was high: mood on 93.8% of the days (872/930), sleep on 89.8% of the days (835/930), activity on 85.6% of the days (796/930), and medication on 88.0% of the days (818/930).

### Mood, Sleep, and Activity Outcomes From the Daybuilder System

**Figure 2:**
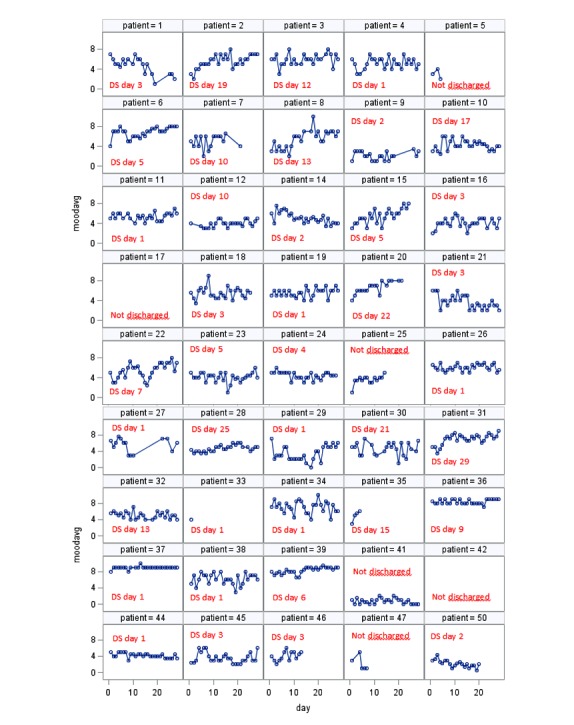
Individual daily self-assessed mood scores with day of discharge (DS) inserted.

Table 3 details the self-assessment scores from the Daybuilder system. Self-assessed mood was not significantly improved during the four-week study period (*P*=.08). Additional analyses showed that the frequency of mood scores <4 (equivalent to severe depression), were prevalent in all three phases: 44% in Phase 1, 31% in Phase 2, and 32% in Phase 3. Figure 2 shows each patient’s self-assessed mood scores from the Daybuilder system, with day of discharge inserted (marked DS in the figure), and illustrates the high day-to-day variability. Inspection of the patients that were readmitted showed no substantial worsening of mood prior to readmission.

**Table 3:** Estimated self-assessment scores from the Daybuilder system.

Day	Mood score (SE)	Sleep-onset hh:mm (SE, min)	Sleep-offset hh:mm (SE, min)	Sleep-midpoint hh:mm (SE, min)	Sleep Quality score (SE)	Activity minutes (SE)
Day 1	4.7 (0.3)	23:30 (9)	7:42 (9)	3:36 (8)	5.2 (0.2)	156.9 (17.6)
Day 8	4.9 (0.2)	23:38 (9)	7:54 (9)	3:46 (8)	5.4 (0.2)	168.6 (15.0)
Day 15	5.0 (0.3)	23:46 (10)	8:07 (10)	3:56 (10)	5.6 (0.2)	180.2 (15.2)
Day 22	5.1 (0.3)	23:53 (15)	8:19 (12)	4:07 (11)	5.8 (0.2)	191.9 (18.0)
Day 28	5.3 (0.3)	24:00 (14)	8:30 (14)	4:15 (13)	5.9 (0.3)	201.9 (21.8)
Change	0.5 (0.3)	00:29 (10)	00:48 (12)	00:39 (10)	0.7 (0.3)	45.1 (25.6)
*P*-value	.08	.006	<.001	<.001	.04	.09

Sleep-offset at endpoint was delayed to 48 minutes (SE 12) compared to baseline sleep-offset (*P*<.001; t=4.0, CL 24.6-74.7) and was mostly prevalent in Phase 2. The range of sleep-onset was from 19:00 to 06:30, and sleep-offset was from 24:00 to 14:30. should be " Sleep duration was 8:12 (hours:minutes) at baseline and 8:30 (hours:minutes) at endpoint (*P*=.10). Sleep quality was significantly improved during the four-week period from 5.2 (SE 0.2) to 5.9 (SE 0.3; *P*=.04; t=2.2; CL 0.05-1.4). Naps were only taken on 6% of study days (56/931). The mean number of awakenings was 1.1 (1.9; range 0-20) per night.

The mean duration of activity was 156.9 minutes at baseline (SE 17.6) and 201.9 minutes at endpoint (SE 21.8; *P*=.09). Explorative analyses on the effect of sleep parameter on self-reported mood showed no significant effect for sleep-onset, sleep-midpoint, or sleep-offset, but sleep quality was significantly positively associated with mood (parameter estimate 0.15, *P*<.001, t=6.9; CL 0.11-0.19). Furthermore, when estimating delay of sleep-onset (with sleep quality included in the model) during the study period, this showed a significant negative effect on mood (combined effect of sleep-onset and the interaction between sleep-onset and day). Thus, a three-hour delay in sleep-onset reduced mood by 0.4 pointscompared to no delay (SE 0.2; *P*=.03; t=2.2; CL 0.04-0.77). Explorative analyses showed no influence of study phase on self-monitored mood and sleep quality (*P*=.93).

### Interview and Self-Assessment Scores From Paper-and-Pen Questionnaires

During the study period, a statistically significant reduction in the degree of depression was seen on all depressions scales, and an increase in scores was observed on the WHO-5 quality of life scale (Table 4). Correlation between HAM-D_17_ and self-reported mood was 0.51 (*P<*.001) at baseline, and 0.44 (*P*=.02) at endpoint. Linear regression showed a negative impact of HAM-D_17_ baseline scores on adherence to sleep and mood registrations (sleep parameters, R^2^=0.18, *P*=.01; mood scores R^2^=0.10, *P*=.07).

**Table 4:** Scores from depression and quality of life scales.

Scale (n)	Baseline n (SD)	Endpoint n (SD)	*P*-value
Hamilton Depression Rating Scale 17 item version (34)	18.0 (6.5)	13.3 (7.3)	<.001
Hamilton Depression Rating Scale 6 item version (34)	9.9 (3.0)	7.1 (3.7)	<.001
Bech-Rafaelsen Melancholia Scale (34)	17.9 (5.7)	13.3 (7.0)	<.001
Major Depression Inventory (33)	27.1 (13.1)	22.2 (12.7)	.006
5-item World Health Organization Well-Being Index (32)	31.3 (22.9)	43.4 (22.1)	<.001

## Discussion

We expected the Daybuilder system to be manageable for this patient category, and that there would be great day-to-day variability in mood and sleep; both of these expectations were confirmed. Based on prior results [11] we expected that a subset of patients would feel that assessment of mood would worsen their mood. However, this was not the case in our study, as only one patient had this experience.

### Principal Results

Based on user reviews, the usability, acceptability, and adherence of the Daybuilder system were found to be good. Sleep onset and offset varied greatly from day-to-day, and the sleep-wake cycle was delayed significantly from baseline to endpoint. Subjective mood scores entered in the Daybuilder system also varied greatly from day-to-day, and demonstrated no improvement from baseline to endpoint.

Considering the short study period, a dropout rate of 24% (11/45) in a four-week study period must be considered as large, and was mainly caused by worsening of depression, although the present study did not reveal the exact causes for terminating self-monitoring. Due to IOA’s referral rules, this patient group is expected to be more severely depressed and unstable than the typical inpatient with depression, and from this background some dropout is to be expected. Development in this area should focus on mechanisms that will keep patients from dropping out, especially when experiencing a deterioration of mood. This goal could be accomplished by implementing alarm systems activated at mood drops, or when assessments are missing for more than one day.

The secondary outcomes showed that patients’ sleep drifted to later in the day after discharge, and that this drift was associated with worsening of mood. This finding, along with high readmittance rates, calls for an improved system with clinician responses based on visual inspection of daily assessments. The results from this study have prompted us to develop a randomized study that focuses on mood scores and also aims to prevent sleep drift by observing sleep data on a daily basis, and contacting patients in cases of sleep drift or sleep irregularity.

### Limitations

Patients referred to IOA probably belong to a more severely depressed subset of inpatients, and thus do not reflect the general group of inpatients with depression. We do not believe that the inclusion and exclusion criteria had any major impact on patients that were included into the study. The most common reason for noninclusion was due to patients being discharged before we could inform them of the project.

The study design, with a single arm, fails to determine the effects that the Daybuilder system has on depression. This information would require a randomized controlled trial. We cannot know from our data whether an active clinician intervention could have prevented dropout and readmission. This approach would require incorporation of timely and active clinician help, and necessitate that patient data is coupled with an automatic system that alerts clinicians when deterioration is detected, and/or that clinicians view patient data daily. Due to the low sample size, negative results could easily be due to lack of power and randomized studies in this field should include far larger sample sizes. Conversely, we cannot rule out spurious positive findings.

ECT treatment is associated with a clinically recognizable retrograde and anterograde amnesia, but this did not seem to influence the use of the Daybuilder system in a significant way. This finding is possibly a tribute to the user-friendliness of the Daybuilder system. The present study design did not allow clinicians to access any day-to-day assessment of patients’ data, meaning that a worsening of patients’ conditions could only be detected once a week, in relation to the planned telephone consultation. This limitation puts the need for clinical intervention into perspective.

Patients were instructed to enter morning and evening mood scores to assess diurnal variation. However, not all patients succeeded in this; thus, mood data reflects different time points (morning or evening) and some of the day-to-day variation is probably caused by diurnal variation. The registered difficulty in entering a mood score more than once per day is probably due to the way that mood data was entered into the Daybuilder system, which used tabs inside the user interface. Caution should also be taken when interpreting mean mood scores, as patients who dropped out most likely experienced a deterioration of mood, thus inducing a bias.

The low correlation between self-assessed mood and HAM-D_17_ scores at baseline (0.51; *P*<.001) and endpoint (0.44; *P*=.02) points to patients reporting aspects of their illness with a different content than the items covered by the Hamilton score. Postpublication analyses from our earlier study, using a similar depression VAS scale (Preskorn) also showed a low correlation (Spearman) of 0.51 (*P*<.001) with HAM-D_17_ scores [27]. However, this finding does not imply that self-assessed mood scores are less valuable than the Hamilton scores, but only that self-assessed scores report other aspects of the depressive illness (such as negative and positive affect), and are possibly more akin to a patient’s own experiences of their condition. We must also consider whether a higher daily mood sampling frequency would be better to track mood fluctuation.

The results from sleep scores confirm that patients with depression had a dysregulated sleep-wake cycle, with large day-to-day variations and a substantial delay in sleep timing when discharged. The finding that a delay in sleep-onset had a negative impact on mood points to the possibility that an intervention to prevent sleep delay could improve mood and theoretically prevent relapse.

It was a surprise that patients’ self-assessed mood scores did not significantly deteriorate in the days after discharge. It was also unexpected that the self-assessed mood scores for those that were readmitted did not deteriorate in the days preceding readmission. These two results call for speculation on whether the mood assessment that was used should be supplemented with assessments more aimed at patient security, such as monitoring of suicidal ideation or by using depression scales. This consideration has prompted us to change our upcoming study, which also uses electronic self-monitoring, such that the wording of self-assessed *mood* is replaced by self-assessed *depression* severity. Through communication with the patients, we will aim at a common understanding of the word *depression*. The Daybuilder system could be improved if it is developed as an app for smartphones or tablets. This progression would eliminate the need for patients to be near a computer, and make data entry more flexible.

### Conclusions

In conclusion, patients were satisfied with the Daybuilder system and study completers had a high adherence to the Daybuilder application. The dropout rate and the gradual delay in sleep emphasize the need for continued clinical support for patients discharged from psychiatric wards, especially concerning sleep guidance.

Improvement of the current Daybuilder system could be done using daily clinician monitoring and daily responses to data entry. This approach could be restricted to patients with suspected suicidal risk or high risk of relapse, such as patients treated with ECT [28]. Additionally, alarms connected to mood ratings could be incorporated into the software to alert clinicians to patients that are deterioration or not registering data. Other improvements include: development of a smartphone app, the use of chat systems, and SMS options to facilitate communication between patients and clinicians. Such an improved full version would add to patient empowerment and autonomy [29,30]. The present study, focusing on usability, highlights the need for interaction between the clinician and the patient.

## Abbreviations

CL: confidence limit
ECT: Electro Convulsive Treatment
HAM-D: Hamilton depression rating scale
IOA: Intensive Outpatient Unit for Affective Disorders
MDI: Major Depression Inventory
MES: melancholia scale
SD: standard deviation
SE: standard error
SMS: short message service
SUS: System Usability Scale
VAS: Visual Analog Scale
WHO-5: 5-item World Health Organization Well-Being Index
